# Complete mitochondrial genome and phylogenetic relationship of ornate threadfin bream, *Nemipterus hexodon* (Perciformes, Nemipteridae)

**DOI:** 10.1080/23802359.2019.1640081

**Published:** 2019-07-13

**Authors:** Yun Zhai, Su-Fang Niu, Ren-Xie Wu, Ben-Ben Miao, Fang Liu, Chun-Xiao Ou

**Affiliations:** a College of Fisheries, Guangdong Ocean University, Zhanjiang, Guangdong, P.R. China;; b Guangdong Leizhou Rare Marine Life National Nature Reserve, Zhanjiang, Guangdong, P.R. China

**Keywords:** *Nemipterus hexodon*, mitochondrial genome, phylogenetic relationship

## Abstract

The complete mitochondrial genome of the ornate threadfin bream, *Nemipterus hexodon*, was first determined by the pairs-walking sequencing in this study. The circular mtDNA molecule was 17,115 bp in size and the overall nucleotide composition of H-stand was A (29.55%), T (27.36%), G (16.08%), and C (27.01%), with a slight bias towards A + T. The complete mitogenome encoded 13 protein-coding genes, 22 tRNA genes, 2 rRNA genes, and 2 non-coding regions (an origin of L-strand replication and a control region). The Bayesian tree supported the phylogenetic position of *N. hexodon*, which provided useful information for phylogenetic relationship in genus *Nemipterus*.

The ornate threadfin bream, *Nemipterus hexodon* (Quoy and Gaimard 1824), belongs to the family Nemipteridae (Perciformes), which is a warm, small to moderate-sized benthic fish, widely distributed in the Indo-West Pacific Ocean, reaching west to the Andaman Sea, east to Solomon Islands, north to the East China Sea, and south to Australia (Russell 1990, p. 37–38). *Nemipterus hexodon* is one of the artisanal and commercial fisheries in the southeastern coast of China by handline and bottom trawl (Liu et al. 2016). It is very common in the coastal fish market in the northern South China Sea and is a popular edible fish species for local people. However, little is known about the fishery resources and fishery biology of *N. hexodon* and its genetic background has not been reported. Here, we first determined the complete mitogenome sequence of *N. hexodon*, which was expected to provide insight into the phylogenetic relationship and genetic resources of the species.

One specimen of *N. hexodon* was collected in May 2010 from Guangdong Leizhou Rare Marine Life National Nature Reserve, Beibu Gulf, the South China Sea (GPS location: 20°39′33″N, 109°44′39″E). It was preserved in 95% ethanol and deposited in Guangdong Ocean University (No. 201003185). Total genomic DNA was extracted from muscle tissue using standard phenol-chloroform method (Sambrook and Russell 1989). The complete mitogenome of *N. hexodon* was obtained by using PCR method, with 15 primer pairs-walking sequencing strategy.

The complete mitochondrial genome of *N. hexodon* was sequenced to be 17,115 bp in length (GenBank accession number: MK978155). It encoded the canonical 37 genes including 13 protein-coding genes, 22 tRNA genes, and 2 rRNA genes, and 2 non-coding regions (an origin of L-strand replication and a control region). The *ND6* and 8 tRNA genes (*tRNA^Gln^*^, Ala,^ *^Asn, Cys, Tyr, Ser, Glu, and Pro^*) were located on L-strand and other genes were transcribed from H-strand. The overall base composition of H-strand was A (29.55%), T (27.36%), G (16.08%), and C (27.01%), with a slight bias towards A + T, which is similar to other threadfin bream (Li et al. 2016; Wu et al. 2016; Wu and Li 2016). Among the 13 protein-coding genes, 12 protein-coding genes started with typical ATG codon, while the *COI* with GTG codon. Also, 5 protein-coding genes (*ND1*, *COI*, *ATPase8*, *ND4L*, and *ND5*) were terminated with TAA codon, *ND6* with TAG, and other remaining ones with incomplete stop codon TA– or T–. There were some overlaps among *ATPase8* and *ATPase6* (10 bp), *ND4L,* and *ND4* (7 bp), *ND5* and *ND6* (4 bp). The 22 tRNA genes ranged from 67 to 75 bp in size. All tRNA genes could form the typical cloverleaf secondary structures except for *tRNA^Ser^*. The two rRNA genes (*12S* and *16S rRNA*) were 994 bp and 1733 bp in size, respectively and were found between *tRNA^Phe^* and *tRNA^Leu^*, separated by *tRNA^Val^*. The origin of L-strand replication (O_L_) located between *tRNA^Asn^* and *tRNA^Cys^* in the WANCY region. The control region (D-loop) was found between *tRNA^Pro^* and *tRNA^Phe^* and was 1357 bp in length, which is shorter than that of *Nemipterus bathybius* (1603 bp) (Wu et al. 2016), but longer than that of *Nemipterus japonicus* (1260 bp) (Li et al. 2016) and *Nemipterus virgatus* (1260 bp) (Wu and Li 2016). Based on the sequence identity analysis of the family Nemipteridae performed in BioEdit version 7.1.9 (Hall 1999), the mitogenome sequence of *N. hexodon* shared 85–86% and 76% identities with that of three *Nemipterus* species (Li et al. 2016; Wu et al. 2016; Wu and Li 2016) and *Scolopsis vosmeri* (Wu et al. 2017), respectively.

The phylogenetic analysis was constructed by MrBayes version 3.2.7 (Huelsenbeck and Ronquist 2001) based on the complete mitogenome sequences of *N. hexodon* and the other 12 species within four families (Lethrinidae, Lutjanidae, Sparidae, and Nemipteridae) using *Labracinus cyclophthalmus* (AP009125) and *Scatophagus argus* (KC790398) as outgroups. The Bayesian tree (Figure 1) showed that *N. hexodon* first gathered with *N. bathybius*, then clustered together with *N. japonicus* and *N. virgatus* and constituted a monophyly in the family Nemipteridae with *S. vosmeri*. They formed a sister-group relationship with other three families. Altogether, the results absolutely supported the phylogenetic position of *N. hexodon* and provided useful information for phylogenetic relationship in genus *Nemipterus*.

**Figure 1.**
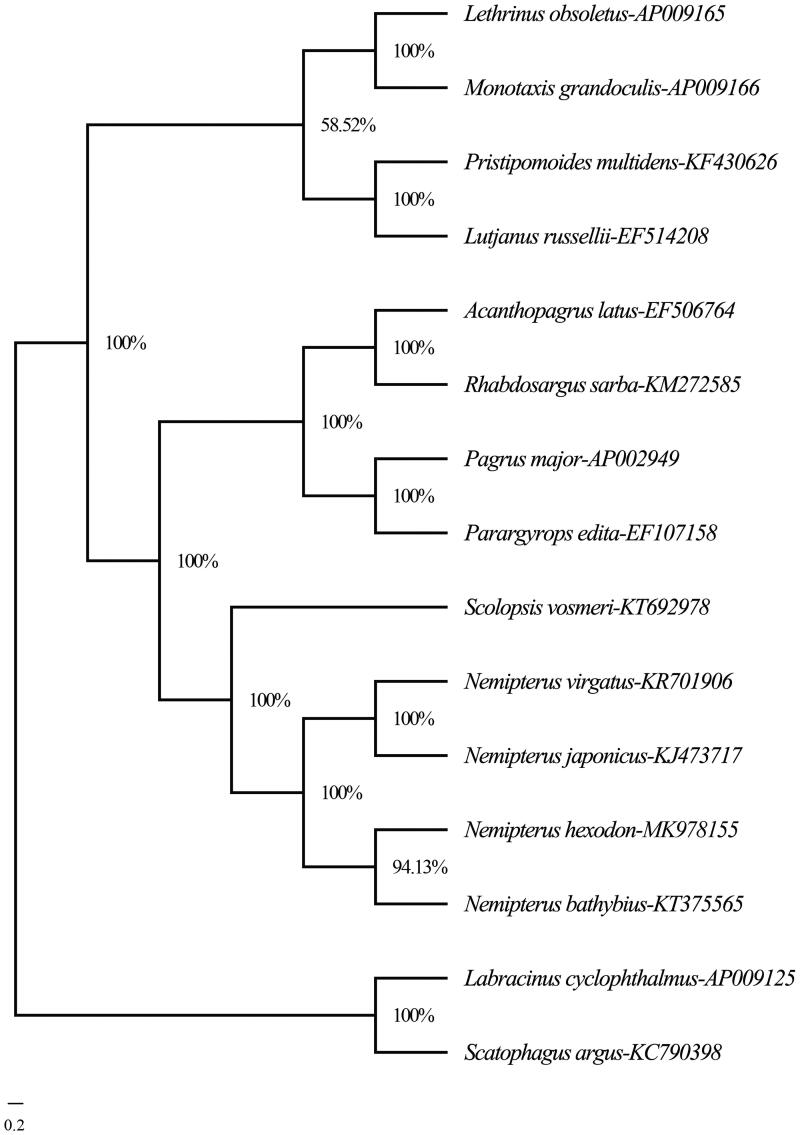
The Bayesian tree of *Nemipterus hexodon* and other 12 species within four families (Lethrinidae, Lutjanidae, Sparidae, and Nemipteridae) based on their complete mitogenome sequences. The bootstrap value was given for each branch.
